# Alcohol Metabolism and Epigenetics Changes

**DOI:** 10.35946/arcr.v35.1.02

**Published:** 2013

**Authors:** Samir Zakhari

**Affiliations:** **Samir Zakhari, Ph.D.,***former director of the Division of Metabolism and Health Effects at the National Institute on Alcohol Abuse and Alcoholism, is Senior Vice President of Science, the Distilled Spirits Council of the United States (DISCUS), Washington, DC.*

**Keywords:** Alcohol consumption, alcohol metabolism, ethanol metabolism, alcohol-induced organ damage, disease, epigenetics, epigenetic mechanisms, epigenetic modifications, gene expression, DNA, DNA methylation, histone modification, histone acetylation

## Abstract

Metabolites, including those generated during ethanol metabolism, can impact disease states by binding to transcription factors and/or modifying chromatin structure, thereby altering gene expression patterns. For example, the activities of enzymes involved in epigenetic modifications such as DNA and histone methylation and histone acetylation, are influenced by the levels of metabolites such as nicotinamide adenine dinucleotide (NAD), adenosine triphosphate (ATP), and S-adenosylmethionine (SAM). Chronic alcohol consumption leads to significant reductions in SAM levels, thereby contributing to DNA hypomethylation. Similarly, ethanol metabolism alters the ratio of NAD^+^ to reduced NAD (NADH) and promotes the formation of reactive oxygen species and acetate, all of which impact epigenetic regulatory mechanisms. In addition to altered carbohydrate metabolism, induction of cell death, and changes in mitochondrial permeability transition, these metabolism-related changes can lead to modulation of epigenetic regulation of gene expression. Understanding the nature of these epigenetic changes will help researchers design novel medications to treat or at least ameliorate alcohol-induced organ damage.

The concept that only DNA and proteins can impact disease states is an oversimplification. It does not take into account different metabolic pathways in which key metabolites bind to transcription factors and alter gene expression patterns that contribute to the observable characteristics (the phenotype) of a given disease. Simple metabolites dictate the actions of specific transcription factors that sense the minute-to-minute cellular environment to determine which parts of, and the extent to which, the genetic code will be transcribed.

An important mechanism in the regulation of gene expression, particularly its first step (transcription), is chromatin remodeling. The human genome is packaged into a dynamic complex of DNA, histone proteins, and non-histone proteins (i.e., chromatin). This chromatin can be packaged more or less densely, and the degree of compactness, which is influenced by histone modifications, determines the DNA’s accessibility to the transcription machinery. In general, condensed chromatin (heterochromatin), which is associated with the removal of acetyl groups (i.e., deacetylation) from histones, mediates transcriptional repression. Conversely, transcriptionally active genes are found in open chromatin areas (i.e., euchromatin).

An example of how simple metabolites affect gene transcription is demonstrated in caloric restriction (CR) studies. Research in yeast and rodents has shown that limiting their caloric intake increases their life span (Anderson and Weindruch 2012). This effect is achieved through internal “sensors” that recognize food scarcity and regulate energy expenditure. One such sensor, the protein encoded by the *silent information regulator2* (*SIR2*) gene in yeast (and its mammalian orthologue *SIRT1*), mediates transcriptional silencing through its nicotinamide adenine dinucleotide (NAD)-dependent histone deacetylase (HDAC) activity. Entire sets of genes may be silenced by NAD-dependent HDACs, thus influencing obesity and longevity following CR. In fact, NAD^+^, reduced NAD (NADH), and the ratio between both compounds (i.e., the NADH/NAD^+^ ratio) all serve important regulatory functions, promoting or preventing numerous biochemical reactions, including gene transcription. Ethanol metabolism can drastically change the NADH/NAD^+^ ratio, providing an example of a metabolic factor that controls gene transcription and thus may influence gene silencing or activation, leading to diseased phenotype.

Many studies suggest that gene expression is not solely determined by the DNA code itself. Rather, gene expression also depends on a host of epigenetic phenomena—that is, gene-regulating activity that does not involve changes to the DNA code. Although the genetic code is the same in all cells, each cell in the body has a unique epigenome that can change over time and which regulates gene expression, thereby determining cellular identity in health. Complex human diseases, which involve feedback between many genes and cells, likely are driven by epigenetic changes and responses as well as by allelic variations. Also, progression of disease often may be better explained by epigenetic alterations than by mutations. Perturbation of cellular epigenetic status (Slomko et al. 2012), for example by alcohol metabolism, can result in the loss of tissue identity or activation of anomalous signaling pathways that lead to developmental defects (e.g., fetal alcohol spectrum disorders [FASD]) or disease states such as liver cirrhosis and cancer. Unlike genetic defects, epigenetic alterations can be reversed by epigenetic therapy.

The best known epigenetic signal is DNA methylation, which tags cytosine, one of the four chemical bases that make up the genetic code, with a methyl group. Cytosine methylation is a major contributor to the generation of disease-causing germline mutations and somatic mutations that cause cancer. Also, abnormal methylation of the promoters of regulatory genes causes their silencing or overexpression—an important pathway to tissue pathology, including cancer. The ability of alcohol to perturb normal patterns of DNA methylation is of considerable interest with regard to its cancer-promoting effects. Global hypomethylation of DNA is a consistent feature of neoplastic transformation.

In addition to DNA methylation, gene expression is influenced by post-translational modifications of histone proteins, such as acetylation, methylation, phosphorylation, ubiquitination, and crotonylation. These modifications determine the genome’s accessibility to transcription factors (Tan et al. 2011). Furthermore, non-coding RNAs (ncRNAs)—including microRNAs (miRNAs), small interfering RNAs (siRNAs), long non-coding RNAs (lncRNAs), promoter-associated RNAs (PARs), and enhancer RNAs (eRNAs)—have emerged as critical factors that control gene expression, cell-cycle regulation, energy metabolism, chromatin architecture, transcription, and RNA splicing (Collins et al. 2010). Dysregulation of ncRNAs, therefore, contributes to various pathological conditions. These epigenetic alterations may substantially change the transcriptional potential of a cell, thereby altering gene expression with outcomes relevant to particular disease phenotypes.

The role of epigenetics in alcohol’s actions has been reviewed by Shukla and colleagues (2008); their publication comprises an extensive background to this article and is highly recommended to the reader. More recently, Mandrekar (2011) discussed the role of epigenetic regulation in alcoholic liver disease. This article focuses on the perturbation of the folate/methionine cycles that influence DNA and histone methylation, as well as on epigenetic processes that are intertwined with alcohol metabolism, namely the increase in NADH/NAD^+^ ratio and the formation of reactive oxygen species (ROS) and acetate, which affects histone acetylation. Alcohol’s epigenetic effect via a host of ncRNAs is beyond the scope of this article. Understanding how alcohol affects these epigenetic processes and how that leads to tissue damage, including FASD and carcinogenesis, is of paramount importance in drug design for the treatment of alcohol-induced disorders.

## Metabolism and Epigenetics

Metabolism produces the energy necessary for various cellular processes. An imbalance between energy intake and expenditure results in the accumulation of nutrients and metabolites, ultimately leading to metabolic diseases. To avoid this, cells constantly are adjusting their metabolic state based on nutrient availability, using extracellular signaling driven by growth factors, hormones, or cytokines. An important feature of metabolic control is the transcriptional regulation of rate-limiting metabolic enzymes, which usually involves epigenetic mechanisms. The ad hoc levels of metabolites, such as NAD^+^, adenine triphosphate (ATP), acetyl-CoA, and S-adenosylmethionine (SAM), and of metabolic hormones such as insulin and leptin, contribute to the temporal control of gene expression. The activities of enzymes involved in epigenetic modifications, such as DNA methyl-transferases (DNMTs), histone acetyltransferases (HATs), HDACs, histone methyltransferases (HMTs), and histone demethylases (HDMs), are regulated, in part, by the concentrations of their required substrates and cofactors (Lee and Workman 2007). Thus, the cell’s metabolic state is tightly integrated in the epigenome and transcriptional regulation.

An example of the close integration of metabolism and epigenetics is CR, in which food intake is reduced by 30 to 50 percent. CR decreases NADH/NAD^+^ ratio and increases the AMP/ATP ratio. These changes are sensed by SIRT1 (which has HDAC activity) and AMP-activated protein kinase (AMPK), respectively. One of SIRT1’s functions is to deacetylate and activate two proteins called forkhead box protein O1 (FOXO1) and peroxisome proliferator-activated receptor-γ coactivator 1α (PGC-1α), a key regulator of energy metabolism. Both of these result in gene expression favoring the synthesis of glucose in the body (i.e., gluconeogenesis) (Vaquero and Reinberg 2009). AMPK directly phosphorylates PGC-1α, which enables SIRT1 to deacetylate and activate PGC-1α (Cantó and Auwerx 2011).

Recent research suggests that chromatin structure is determined, to a great extent, by metabolic signals, and cells’ decision to proliferate, differentiate, migrate, or be quiescent is determined by their micro-environment (Lu and Thompson 2012). Studies in cancer development have revealed a tight link between metabolism and epigenetics. The molecular connections between metabolism and epigenetic modifications of chromatin (Lu and Thompson 2012) and the role of histones as metabolic sensors that convert changes in metabolism into stable patterns of gene expression (Katada et al. 2012) have been described. The relationship between epigenetic modifications and metabolism is complex and bidirectional. On the one hand, epigenetic changes can influence metabolism by regulating the expression of metabolic enzymes (Wolf et al. 2011). On the other hand, metabolism can disturb epigenetic status, resulting in changes in gene expression or chromatin structure (Wellen and Thompson 2012). In fact, all epigenetic enzymes depend on various substrates or cofactors that are intermediates of cell metabolism (Locasale and Cantley 2011).

The molecular machinery that senses changes in the micro-environment of cells and translates them into epigenome modulations of DNA or histone tails, thereby influencing gene expression, is comprised of various kinases, acetyltransferases, and methyltransferases. These enzymes need appropriate levels of phosphate, acetyl, and methyl groups, respectively, to elicit these modifications (Lee and Workman 2007). As a result, chromatin-remodeling enzymes consume key metabolites such as SAM for methylation, ATP for phosphorylation, and acetyl-CoA, NAD^+^, NADH, and acetyl-ADP-ribose for acetylation. These enzymes differ significantly in their affinities for their cofactors, which together with fluctuations in the cofactors’ concentrations and their subcellular distribution (e.g., as a result of circadian rhythmicity, oxygen tension, or nutritional status) influence enzymes’ ability to perform their functions (Albaugh et al. 2011). At least two groups of chromatin regulators—the sirtuins that act as class III histone deacetylases and the poly-ADP ribose polymerases (PARPs)—depend on NAD^+^ levels, which are regulated in a circadian manner linked to energy metabolism (Nakahata et al. 2009). Many signaling pathways, such as Notch and TGFβ, in conjunction with downstream transcription factors, can express or recruit enzymes that modify chromatin (Mohammad and Baylin 2010).

### DNA Methylation

**Figure 1:**
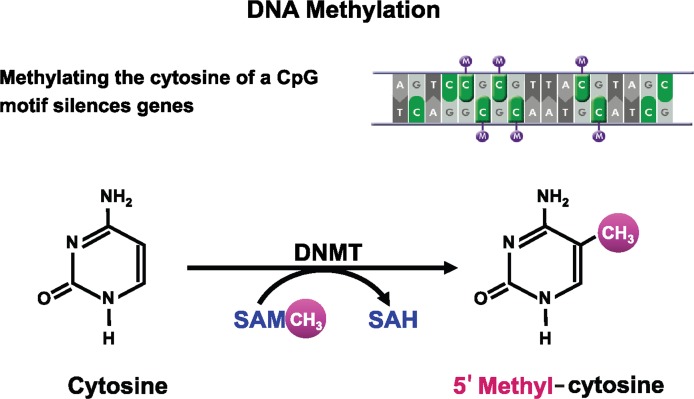
Schematic representation of DNA methylation, which converts cytosine to 5′methyl-cytosine via the actions of DNA methyltransferase (DNMT). DNA methylation typically occurs at cytosines that are followed by a guanine (i.e., CpG motifs). NOTES: SAM = S-adenosylmethionine; SAH = S-adenosylhomocysteine.

The cytosine moiety of cytosine–guanine (CpG) dinucleotides in mammalian DNA can be methylated at carbon 5 to form 5′ methyl-cytosine (figure 1). The methyl group for this chemical modification of the DNA is donated by SAM. This reaction is catalyzed by a family of DNMTs. Of these, DNMT3A and 3B primarily perform de novo methyl transfer, whereas DNMT1 mainly acts as maintenance DNMT with greater affinity for partially methylated (i.e., hemi-methylated) DNA. Methylation of cytosine in CpG-rich regions (i.e., CpG islands) located in or near gene promoters results in gene silencing (Ulrey et al. 2005).

**Figure 2:**
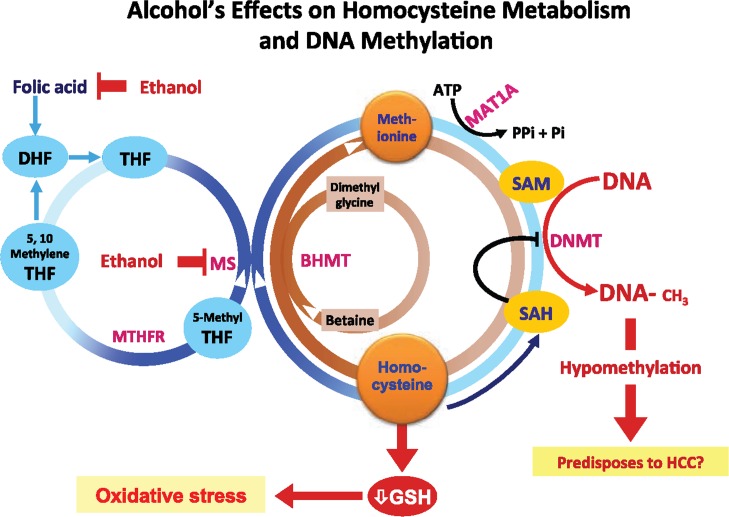
Alcohol’s effects on homocysteine/methionine metabolism and DNA methylation. Methionine, which is formed by methylation of homocysteine (using either 5-methyl tetrahydrofolate [5-methyl THF] or betaine as methyl donors), is essential for the production of S-adenosylmethionine (SAM), which in turn is used to methylate DNA. Chronic heavy drinking reduces folate levels and inhibits methionine synthase (MS), resulting in the reduction of methionine and SAM and the concurrent increase in homocysteine and S-adenosylhomocysteine (SAH). SAH further inhibits DNA methyltransferases (DNMTs), ultimately resulting in global hypomethylation of DNA. NOTES: MTHFR = methylene tetrahydrofolate reductase; MAT = methionine adenosyltransferase; HCC = hepatocellular carcinoma; BHMT = betaine homocysteine methyltransferases; GSH = glutathione; ATP = adenosine triphosphate; Pi = inorganic phosphate.

SAM, a methyl donor for reactions catalyzed by DNMT, is generated by adding ATP to methionine by the enzyme methionine adenosyl transferase (MAT) (figure 2). After the methyl transfer reaction, SAM forms a byproduct, S-adenosyl homocysteine (SAH), which acts as a potent inhibitor of DNMT and HMTs. SAH then is broken down by SAH hydrolase (SAHH) to form homocysteine, which can either enter a set of reactions called the transsulfuration pathway to form glutathione (GSH) or be remethylated to form methionine (Grillo and Colombatto 2008). For remethylation of homocysteine, a methyl group can be transferred either from N5-methyl tetrahydrofolate (THF) by methionine synthase, or from betaine by betaine homocysteine methyl transferase (BHMT). Excessive ROS formation, which can occur during ethanol metabolism, acutely can deplete GSH. This could promote the transsulfuration of homocysteine to generate new GSH and thus divert the reactions from producing methionine and SAM, thereby decreasing DNA methylation.

Chronic alcohol consumption leads to substantial DNA hypomethylation as a result of significant reduction in tissue SAM. Rats fed alcohol for nine weeks showed a decrease in hepatic concentrations of SAM, methionine, and GSH as well as about 40-percent reduction in methylation (Lu et al. 2000). In addition, the investigators observed increased expression of a gene called *c-myc* and increased accumulation of breaks in the DNA strand, both of which predispose to hepatocellular carcinoma (HCC). In fact, chronic hepatic SAM deficiency in a certain mouse strain (i.e., Mat1a knockout mice) resulted in spontaneous development of HCC (Martinez-Chantar et al. 2002). Additionally, alcohol perturbs the folate cycle that is involved in methionine production and the generation of DNA building blocks (i.e., purines and pyrimidines) and which is integral to supplying the methyl groups necessary for DNA methylation.

Altered methionine metabolism and the subsequent hypomethylation is one mechanism by which alcohol produces alcoholic liver disease and HCC (Medici and Halsted 2013). In addition, alcohol-induced degradation of DNMTs and hypomethylation supports a potential epigenetic mechanism for FASD (Chen et al. 2013; Mukhopadhyay et al. 2013).

### Histone Modification

Histone modification plays an important part in epigenetics, affecting transcription, DNA repair, and DNA replication (Esteller 2008). As mentioned above, histone modifications include a plethora of post-translational modifications. This review, however, focuses only on histone acetylation and methylation.

Histone acetylation is regulated mainly by the opposing activities of two families of enzymes—the HATs that acetylate histones and the HDACs (Shahbazian and Grunstein 2007). HATs, which transfer acetyl groups from acetyl-CoA to lysine residues, include three main subfamilies that are functionally distinct—GCN5-related N-acetyltransferase (GNAT), MYST histone acetyltransferase, and p300/CBP. HDACs, in contrast, remove acetyl groups from histones; they comprise four groups (classes I–IV) (Zhang and Dent 2005), some of which are dependent on Zn2^+^ (Haberland et al. 2009). Class III HDACs, known as sirtuins, however, require NAD^+^ as a cofactor. In general, histone acetylation results in transcriptional activation, whereas deacetylation is associated with gene silencing (Lane and Chabner 2009).

Histone methylation is achieved by HMTs. They can be classified into three groups: SET domain and non-SET domain lysine methyltransferases, and arginine methyltransferases. All of these use SAM as a coenzyme to transfer methyl groups to lysine or arginine residues of substrate proteins. There are three distinct states of lysine methylation (i.e., mono-, di-, and tri-methylated) (Varier and Timmers 2011). Histone methylation can result in transcriptional activation or repression, depending on the position of the lysine that is modified (Berger 2007). For example, methylation of H3K4,^1^ H3K36, and H3K791 is associated with active transcription, whereas methylation of H3K9, H3K27, and H4K20 generally indicates silenced chromatin. Histone demethylation is achieved by a group of enzymes collectively known as HDMs.

**Figure 3:**
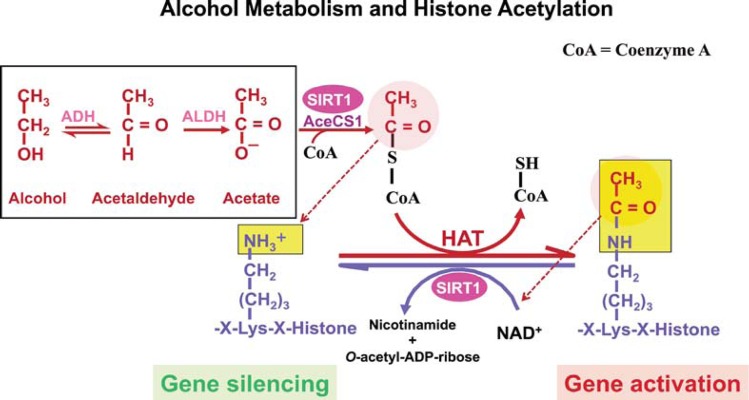
Alcohol metabolism and histone acetylation. Acetyl-coenzyme A (acetyl-CoA) synthetase (AceCS), an enzyme that converts acetate to acetyl-CoA, is activated by SIRT1. Acetyl-CoA is used by histone acetyltransferase (HAT) to acetylate the lysine residues in histone proteins. This neutralizes the positive charge and allows the chromatin to assume an open conformation, thus resulting in gene activation. SIRT1 also deaceytlates acetylated histones, resulting in gene silencing. Thus, SIRT1 is a sensor that balances gene activation and silencing in the cell based on the cell’s energy status. Alcohol metabolism results in acetate formation, which is used in extrahepatic tissues to produce acetyl-CoA. NOTES: AceCS1 = Acetyl-CoA synthase 1; ADH = alcohol dehydrogenase; ALDH = Aldehyde dehydrogenase.

The effects of alcohol metabolism on histone acetylation have been demonstrated in animal experiments, including studies of obese mice.^2^ Alcohol administration to these animals was associated with exacerbation of fatty liver, which resulted from an impairment of the hepatic lipid metabolism pathways, mainly those mediated by SIRT1 and AMPK (Everitt et al. 2012). The development of alcohol-induced fatty liver could be prevented by administering rosiglitazone, an anti-diabetic medication that binds to certain receptors in fat cells and makes them more sensitive to insulin. The protective effect of rosiglitazone was attributed to its enhancement of the hepatic adiponectin–SIRT1–AMPK signaling pathway (Shen et al. 2010). Other studies found that chronic alcohol consumption can result in protein hyper-acetylation within cell components called mitochondria. Most proteins in the mitochondria normally are deacetylated through SIRT3-dependent mechanisms (Fritz et al. 2012). Ethanol-induced suppression of SIRT3 and the concomitant increase of another acetylation pathway (i.e., cyclophilin-D acetylation) could be prevented by AMPK activation (Shulga and Pasorino 2010). The role of alcohol metabolism in histone acetylation is shown in figure 3.

## Alcohol Metabolism and Its Effects on Epigenetic Mechanisms

To appreciate the effects of alcohol on histone acetylation and changes in redox state that result in epigenetic modifications of gene expression, a brief overview of alcohol metabolism is warranted.

Alcohol is metabolized mainly by two pathways—oxidative pathways that take place mainly in the liver and non-oxidative pathways that occur mainly in extrahepatic tissues. The following discussion will focus only on oxidative pathways.

Oxidative ethanol metabolism mainly occurs in the liver via a major pathway in which the enzyme cytosolic alcohol dehydrogenase (ADH) produces acetaldehyde, a highly reactive and toxic molecule. This oxidation is accompanied by the reduction of NAD^+^ to NADH. Through this pathway, ethanol oxidation generates a highly reduced cytosolic environment, predominantly in liver cells (i.e., hepatocytes).

**Figure 4:**
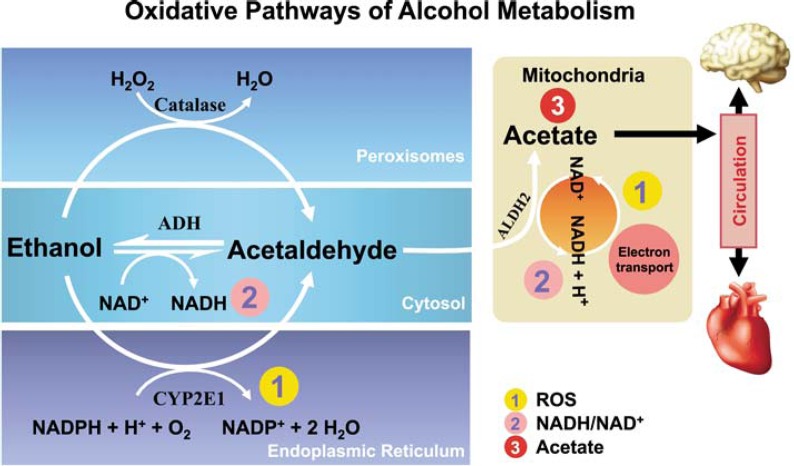
Oxidative pathways of alcohol metabolism. Alcohol is metabolized mainly in the cytosol by alcohol dehydrogenase (ADH) to produce acetaldehyde. At high levels of alcohol consumption, an enzyme in the endoplasmic reticulum, cytochrome P450 IIE1 (CYP2E1), becomes involved in metabolizing alcohol to acetaldehyde; this enzyme also is induced by chronic drinking. A catalase-mediated reaction in the peroxisomes is considered a minor metabolic pathway of alcohol metabolism. Acetaldehyde is further metabolized to acetate in the mitochondria. Alcohol metabolism results in the formation of NADH and thus changes the redox state of hepatocytes (i.e., increases the ratio of NADH/NAD^+^). Both alcohol metabolism by CYP2E1 and the re-oxidation of NADH via the electron transport chain in the mitochondria results in the formation of reactive oxygen species (ROS).

In addition to ADH, a group of enzymes known as the cytochrome P450 isozymes, including CYP2E1, 1A2, and 3A4, also contribute to ethanol oxidation to acetaldehyde in the liver. These enzymes, which are present predominantly in a cell component called the endoplasmic reticulum (ER), become involved particularly after chronic ethanol intake. CYP2E1 is induced by chronic ethanol consumption and assumes an important role in metabolizing ethanol to acetaldehyde at elevated alcohol concentrations.^3^ Alcohol metabolism by CYP2E1 also produces highly reactive ROS, including hydroxyethyl, superoxide anions, and hydroxyl radicals. Finally, another enzyme called catalase, which is located in cell components called peroxisomes, also can oxidize ethanol (figure 4); however, quantitatively this is considered a minor pathway of ethanol oxidation. All of these oxidative pathways generate acetaldehyde, which then is rapidly metabolized further. This is done mainly by mitochondrial aldehyde dehydrogenase (ALDH2) to form acetate and NADH. Mitochondrial NADH is oxidized by the electron transport chain.

## Epigenetics-Relevant Consequences of Oxidative Alcohol Metabolism

Oxidative alcohol metabolism can exert epigenetic effects through several mechanisms, including increase in the NADH/NAD^+^ ratio and the formation of ROS and acetate.

### Increases in NADH/NAD^+^ Ratio and Their Consequences

The ratio of NADH to NAD^+^ fluctuates in response to changes in metabolism. Alcohol metabolism produces a significant increase in the hepatic NADH/NAD^+^ ratio in the cytoplasm and mitochondria of hepatocytes, as evidenced by changes in the levels of several other molecules in those cell compartments (i.e., an increase in the lactate/pyruvate ratio in the cytoplasm and in the γ-hydroxybutyrate/acetoacetate ratio in the mitochondria) (Cunningham and Bailey 2001). The resulting shift of the redox potential of the hepatocytes causes a marked alteration in various reversible metabolic pathways (Krebs and Veech 1970). As a result, ethanol oxidation vastly increases the availability of oxidizable NADH to the electron transport chain in the mitochondria. NAD^+^ influences many important cellular reactions.

NAD^+^ and NADH mainly are used by enzymes that catalyze substrate oxidation involving energy metabolism, histone deacetylation, and cell death. Under normal physiological conditions, the ratio of cytosolic free NAD^+^ to NADH is approximately 700:1, whereas that of the mitochondria has been reported to be 7–8 to 1 (Stubbs et al. 1972; Veech et al. 1969). Increased NADH/NAD^+^ ratios (e.g., because of depletion of NAD^+^) in both the cytosol and mitochondria of hepatocytes influence the direction of several reversible reactions, leading to alterations in hepatic lipid, carbohydrate, protein, lactate, and uric acid metabolism. The increase in the ratio of NADH/NAD^+^ also results in derangement of carbohydrate metabolism, cell death, modulation of the mitochondrial permeability transition (MPT) opening, and modulation of gene expression.

#### Derangement of Carbohydrate Metabolism

NAD^+^ mediates cytosolic energy metabolism by influencing the breakdown of glucose molecules (i.e., glycolysis) and by modulating the lactate–pyruvate conversion by lactate dehydrogenase (LDH). An increase in the NADH/NAD^+^ ratio interferes with both of these processes. NAD^+^ depletion also causes inhibition of the later steps of glucose metabolism (i.e., the tricarboxylic acid [TCA]) cycle. As NADH accumulates, NAD^+^ becomes depleted. As a result, oxidation of acetyl-CoA by the TCA cycle is inhibited because of a lack of oxidized coenzymes. In addition, NADH accumulation inhibits pyruvate dehydrogenase (PDH), thus decreasing the conversion of pyruvate to acetyl-CoA. Instead, NADH accumulation in the cytosol favors the conversion of pyruvate to lactate by LDH. This lowers the concentration of pyruvate, which in turn decreases the pyruvate carboxylase reaction, one of the rate limiting steps of gluconeogenesis (Krebs et al. 1969). Collectively, the increase in NADH results in the inhibition of gluconeogenesis and, during starvation, can cause clinically significant hypoglycemia.

#### Cell Death

Mitochondria play important roles in the regulation of cell death (i.e., apoptosis and necrosis). They release pro-apoptotic factors such as cytochrome c and apoptosis-inducing factor (AIF), which activate caspase-dependent and caspase-independent cell death, respectively. Another enzyme called poly(ADP-ribose) polymerase 1 (PARP-1), a mediator of programmed necrosis activated by oxidative stress, is an important activator of caspase-independent cell death (Zhang et al. 1994). Overactivation of PARP-1 can induce NAD^+^ depletion (Ying et al. 2003), leading to the inhibition of SIRT1 as well as inhibition of glycolysis, which in turn would reduce pyruvate supply to the TCA cycle and cause ATP depletion (Ying et al. 2002). The NADH/NAD^+^ ratio also affects MPT (Alano et al. 2004), which results in the translocation of AIF from mitochondria to the nucleus (Churbanova and Sevrioukova 2008; Yu et al. 2002), ultimately resulting in apoptosis. Thus, an increased NADH/NAD^+^ ratio caused by alcohol metabolism can influence pro-death and pro-survival signals in the PARP-1–mediated cell-death program.

#### Modulation of the MPT Opening

MPT is defined as an increase in the ability of the membranes surrounding the mitochondria to allow passage of molecules of a certain size (i.e., increase in membrane permeability). This increase in permeability, which results from the opening of specific pores, typically occurs in response to certain pathological conditions and can lead to mitochondrial swelling and cell death through apoptosis or necrosis. Increases in NADH/NAD^+^ ratio resulting from ethanol metabolism lead to an increase in MPT opening, which significantly influences mitochondrial membrane potential (Zoratti and Szabo 1995). In addition, NADH can have other effects, such as increased release of calcium ions (Ca^++^) release from certain channels on the ER, as demonstrated in a cell line called PC12 cells (Kaplin et al. 1996), and inhibition of ryanodine receptors of cardiac muscles (Zima et al. 2003).

#### Modulation of Gene Expression

The NADH/NAD^+^ ratio influences gene expression through several pathways. The following discussion focuses on three NAD^+^-dependent enzymes: carboxyl-terminal binding protein (CtBP); silent information regulator (Sir2); and the heterodimeric Clock/NPAS2 transcriptional regulator, whose activities may play a role in ethanol-induced injury.

CtBP is a regulatory factor mediating transcriptional repression that is important for cell cycle regulation and development; it acts as a NAD^+^-dependent D2-hydroxyacid dehydrogenase (Kumar 2002). Studies on mice carrying altered *Ctbp* genes suggest that two mutant genes (*Ctbp1* and *Ctbp2*) play unique regulatory roles during development (Chinnadurai 2003). Mice lacking a functional *Ctbp1* gene (i.e., *Ctbp1*-null mice) are about 30 percent smaller than wild type mice, whereas *Ctbp2*-null mice exhibit defects in heart morphogenesis and neural structures. NAD^+^ enhances the interaction of CtBP with target transcription factors (Zhang et al. 2002). Furthermore, CtBP is a metabolic sensor that has been implicated in regulating adipogenesis (Jack et al. 2011). The role of CtBP and changes in the redox state caused by alcohol metabolism in ethanol-induced teratogenesis and effects on adipose tissue remains to be elucidated.

The Sir2 protein links cellular metabolism and transcriptional silencing through its NAD^+^-dependent HDAC activity (Imai et al. 2000). This activity is essential for Sir2 functions, including gene silencing, regulation of the circadian clock, and its role in obesity and longevity (Rutter et al. 2001). The extraordinary requirement of NAD^+^ in the deacetylation reaction suggests that Sir2 may function as a sensor for the energy status of cells, linking the energy level represented by available NAD^+^ to the silencing of gene expression. The mammalian orthologue of Sir2, known as SIRT1, also is a NAD^+^-dependent deacetylase (Imai et al. 2000) whose substrates include histones and the transcription factor p53 (Vaziri et al. 2001). NAD^+^ activates Sir2 during CR, which not only extends the life span in a wide variety of organisms, but also reduces the incidence of age-related diseases such as diabetes, cancer, immune deficiencies, and cardiovascular disorders (Bordone and Guarente 2005).

The Clock protein is part of the transcriptional feedback system whose activity fluctuates with the light–dark cycle and which controls the circadian rhythm in mammals. Generally, humans work, eat, and exercise during the day and rest at night. Synchronization of these activities with metabolic reactions is achieved by the circadian clock. The circadian system comprises a central clock, which is regulated by light and located in a brain region called the suprachiasmatic nucleus (SCN) of the anterior hypothalamus, and peripheral clocks present in metabolic tissues that are entrained by the central clock via feeding/fasting cycles. The master regulators (transcriptional activators) of the central clock are two proteins called circadian locomotor output cycles kaput (CLOCK) and brain and muscle ARNTL-like protein 1 (BMAL1). Both of these are transcriptional factors that regulate the expression of cryptochrome (*CRY1* and *CRY2*) and period (*PER1, PER2, PER3*) genes. *PER* and *CRY* proteins bind to CLOCK/BMAL1 and inhibit their transcriptional activity (Bass and Takahashi 2010). Furthermore, using the nuclear receptor REV-ERB as a feedback loop, BMAL1 drives the transcription of *Rev-erb*α, which in turn inhibits *Bmal1* transcription (Preitner et al. 2002). In addition, the retinoid-related orphan receptors (RORα, β, γ) activate BMAL1 and REV-ERBα (Jetten 2009). Misalignment of these activities with the internal clock disrupts energy homeostasis and could result in metabolic diseases, such as those observed in shift workers (Wang et al. 2011).

Conversely, metabolic reactions and the resulting redox state of cells have been shown to play an important role in the function of the circadian rhythm. For example, the intracellular NADH/NAD^+^ ratio, through the sensor SIRT1, influences BMAL1 and CLOCK in both central and peripheral clocks. In addition, AMP levels in the cell regulate the circadian clock by activating AMPK through inhibiting AMPK dephosphorylation (Um et al. 2011). Thus, whereas the central clock is regulated by an environmental cue to the SCN (i.e., light sensed by the retina), cellular metabolites influence the peripheral clocks, which are also entrained by the central clock.

Human and animal studies have demonstrated that both acute and chronic alcohol intake can affect many aspects of circadian rhythms, including physiological, endocrine, and behavioral functions. Alcohol intake and withdrawal have been shown to affect the circadian rhythm of body temperature in rats and to alter circadian melatonin secretion in both healthy and alcoholic people (Danel and Touitou 2006; Danel et al. 2009). In addition, alcohol alters the circadian expression of *Per2* and *Per3* in the SCN, suggesting that alcohol may directly affect the central pacemaker and interfere with its circadian functioning (Chen et al. 2004). In rats, Farnell and colleagues (2008) have demonstrated that neonatal alcohol exposure during the brain growth spurt can alter clock gene oscillations in the liver, in addition to the SCN.

### Formation of ROS and Oxidative Stress and Their Consequences

ROS, including superoxide (• O_2^−^_), hydrogen peroxide (H_2_O_2_), hypochlorite ion (OCl^−^), and hydroxyl (• OH) radicals, are generated by many reactions in multiple compartments in the cell, including NADPH oxidases, lipid metabolism within the peroxisomes; and various cytosolic cyclooxygenases. However, in most cells the vast majority of ROS result from electron transport by the mitochondria. The role of ROS in inducing epigenetic alterations in human carcinogenesis has been discussed (Ziech et al. 2011).

## Acetate Formation and Its Consequences

Most of the acetate resulting from ethanol metabolism escapes the liver into the blood. In cells with mitochondria that contain enzymes capable of transforming acetate to acetyl CoA, such as heart, skeletal muscle, and brain, the acetate is eventually metabolized to CO_2_ in the TCA cycle. As shown in figure 3, SIRT1 activates mammalian acetyl-CoA synthase through deacetylation, resulting in the formation of acetyl-CoA. The acetyl-CoA then is used to acetylate histones, resulting in gene activation. Subsequently, SIRT1 deacetylates the histones, resulting in gene silencing. Thus, SIRT1 can act as a sensor to regulate gene transcription.

## Summary and Conclusions

**Figure 5:**
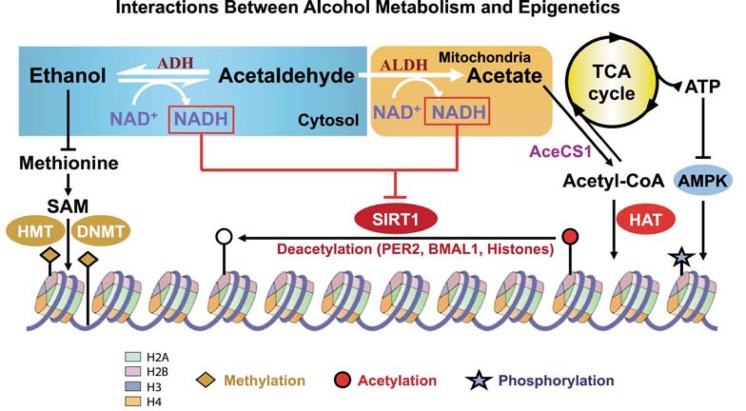
Interactions between alcohol metabolism and epigenetic mechanisms. Chronic alcohol consumption leads to lower-than-normal methylation (i.e., hypomethylation) by decreasing the levels of S-adenosylmethionine (SAM), which is used by DNA methyltransferases (DNMTs) and histone methyl transferases (HMTs) to methylate DNA and histones, respectively. Furthermore, alcohol metabolism increases the ratio of the reduced nicotinamide adenine dinucleotide (NADH) to the oxidized nicotinamide adenine dinucleotide (NAD^+^); this inhibits SIRT1, thereby interfering with normal histone acetylation patterns. NOTES: ATP = Adenosine triphosphate; AMPK = AMP-activated protein kinase; HAT = histone acetyl transferase; TCA = tricarboxylic acid cycle.

Figure 5 summarizes the epigenetic effects of alcohol metabolism, which include the following:

- Global hypomethylation resulting from a reduction in SAM levels. SAM levels are reduced as a result of alcohol-induced reduction in folate and the inhibition of methionine synthase. At the same time, SAH levels increase, which inhibits DNMT.
- Histone modification that is associated with an increase in NADH levels caused by alcohol metabolism. The increase in NADH affects SIRT1 activity, leading to gene expression and/or silencing.
- Production of ROS, which affect the expression of inflammatory genes, and acetate, which is used in extrahepatic tissues to produce acetyl-CoA. The latter then is used in histone acetylation by HATs.

These epigenetic changes resulting from chronic alcohol consumption can lead to organ pathology. Understanding the exact nature of the epigenetic changes will help design medication for the treatment or alleviation of alcohol-induced organ damage.

## Footnotes

1 Histone modifications are denoted by the number of the histone protein affected (e.g., histone 3 is noted as H3) as well as the lysine residue affected (e.g., the fourth lysine in the molecule is denoted as K4).

2 The animals had inherited two gene copies predisposing them to obesity (i.e., had an ob/ob genotype).

3 In other words, CYP2E1 has a high Km for ethanol of 8–10 mM, compared with a Km of 0.05–40 mM for hepatic ADH.
